# To form or not to form: PuO_2_ nanoparticles at acidic pH†

**DOI:** 10.1039/d1en00666e

**Published:** 2022-03-11

**Authors:** Evgeny Gerber, Anna Yu. Romanchuk, Stephan Weiss, Anastasiia Kuzenkova, Myrtille O. J. Y. Hunault, Stephen Bauters, Alexander Egorov, Sergei M. Butorin, Stepan N. Kalmykov, Kristina O. Kvashnina

**Affiliations:** Lomonosov Moscow State University, Department of Chemistry 119991 Moscow Russia; The Rossendorf Beamline at ESRF – The European Synchrotron CS40220 38043 Grenoble Cedex 9 France kristina.kvashnina@esrf.fr; Helmholtz Zentrum Dresden-Rossendorf (HZDR), Institute of Resource Ecology PO Box 510119 01314 Dresden Germany; Synchrotron SOLEIL, L'Orme des Merisiers Saint Aubin BP 48 91192 Gif-sur-Yvette France; Condensed Matter Physics of Energy Materials, X-ray Photon Science, Department of Physics and Astronomy, Uppsala University P.O. Box 516 SE-751 20 Uppsala Uppsala Sweden

## Abstract

The aim of this study is to synthesize PuO_2_ nanoparticles (NPs) at low pH values and characterize the materials using laboratory and synchrotron-based methods. Properties of the PuO_2_ NPs formed under acidic conditions (pH 1–4) are explored here at the atomic scale. High-resolution transmission electron microscopy (HRTEM) is applied to characterize the crystallinity, morphology and size of the particles. It is found that 2 nm crystalline NPs are formed with a PuO_2_ crystal structure. High energy resolution fluorescence detected (HERFD) X-ray absorption spectroscopy at the Pu M_4_ edge has been used to identify the Pu oxidation states and recorded data are analysed using the theory based on the Anderson impurity model (AIM). The experimental data obtained on NPs show that the Pu(iv) oxidation state dominates in all NPs formed at pH 1–4. However, the suspension at pH 1 demonstrates the presence of Pu(iii) and Pu(vi) in addition to the Pu(iv), which is associated with redox dissolution of PuO_2_ NPs under acidic conditions. We discuss in detail the mechanism that affects the PuO_2_ NPs synthesis under acidic conditions and compare it with one in neutral and alkaline conditions. Hence, the results shown here, together with the first Pu M_4_ HERFD data on PuF_3_ and PuF_4_ compounds, are significant for the colloid facilitated transport governing the migration of plutonium in a subsurface environment.

## Abstract 2

The combination of advanced spectroscopic and microscopic methods used in this work enables molecular and atomic levels understanding of the Pu(iv) nanoparticles formation under acidic conditions (pH 1–4).

## Graphical abstract

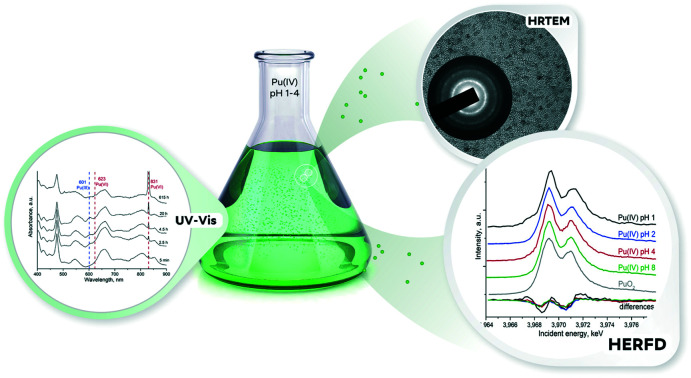

**Environmental significance**

Plutonium is one of the most toxic elements that was ever released to the environment due to the human activities in the field of nuclear weapons production and testing as well as peaceful nuclear energy applications. It has a very complex chemistry and migration behaviour as it has at least four oxidation states at environmentally relevant conditions and a strong tendency to form precipitates and colloids. Stepwise formation of polynuclear species and nanoparticles because of hydrolysis of Pu(iv) is an important process that governs its distribution in subsurface environments. However, a knowledge gap remains concerning the mechanisms of nanoparticle formation from polynuclear Pu(iv) hydrolyzed species that occurs at low pH values. The combination of various advanced spectroscopic and microscopic methods used in this work enables molecular and atomic levels understanding of the chemistry behind Pu(iv) nanoparticle formation.

## 1. Introduction

Plutonium (Pu) is undoubtedly one of the most puzzling elements of the periodic table. Pu may exist in oxidation states III, IV, V, VI and VII, though the latter is relatively stable only under alkaline oxidizing conditions. The most prominent peculiarity of Pu chemical complexity is the ease of redox transformations between Pu(iii), Pu(iv), Pu(v) and Pu(iv), which allows Pu to exist under certain conditions in all four oxidation states simultaneously, even in natural waters. Being one of the primary components of the spent nuclear fuel and nuclear wastes in terms of long-term radiotoxicity, Pu draws attention not only from a fundamental point of view, but also from environmental applied science. The pH of acidic soils is in the range of 3.5–6.5 though ultra-acidic soils can be even lower; high-level waste from the reprocessing of spent nuclear fuel is also highly acidic.^1–4^ Besides that, under low pH the interactions of Pu with humic and fulvic acids are also investigated under low pH (1–4).^5,6^

Plutonium in the environment can be present in various species, depending on the conditions. In the solution it may exist in the form of an ion, either hydrated or with other ligands; it also can be present as a product of polymerization reaction in the form of mono-/polynuclear species. PuO_2_ NPs are part of the crucial Pu species in terms of their environmental behaviour. Despite the fact that PuO_2_ solubility is extremely low, Pu has been shown to migrate with mineral^3,7^ or organic colloids,^8^ therefore, colloid-facilitated transport might play an important role in the transporting of plutonium.^9^ The formation of PuO_2+*x*_ NPs during the reactions at solid–liquid interfaces ranging from natural minerals^10–14^ to microorganisms^15,16^ has been reported.

For any processes involving Pu, its solubility should be taken into consideration. Pu(iv) is extremely prone to hydrolysis which is accompanied by the formation of mono- and oligomeric species. While the solubility of Pu in neutral and alkaline conditions is known to be exceedingly low,^17,18^ the Pu concentration in solution significantly increases in acidic conditions. Redox potential and pH are decisive solution parameters for the solubility and oxidation state distribution of Pu.^19^ Pérez-Bustamante^20^ and Rai with coworkers^21^ have found that Pu(iv) solubility increases due to the aqueous species of other Pu oxidation states arising from Pu(iv) oxidation and disproportionation reactions. Kim and Kanellakopulos^22^ showed that Pu(iv) colloid formation may interfere even at pH 0–1, and it is extremely challenging to successfully separate them from solution even with ultrafiltration. Though, extremely small and stable Pu NPs can still be present in the solution and affect solubility and redox processes.

The presence of very small Pu colloids or polynuclear species can lead to enhancement of apparent solubility by up to 2 orders of magnitude, depending on pH. The composition of these colloids and polymers have been investigated by electrospray time-of-flight mass-spectrometry (ESI-TOF-MS),^23^ laser-induced breakdown detection (LIBD)^23,24^ and extended X-ray absorption fine structure (EXAFS) spectroscopy.^23–25^ The presence of oxo-hydroxo polymers, as well as the presence of Pu(iii) and Pu(v), have been claimed and different mechanisms responsible for these processes were considered.^23–29^ Moreover, it is suggested that Pu(iv) colloids and polymers are in equilibrium with dissolved Pu(iv) and Pu(v). These colloids and polymers also take part in the Pu(iv) to Pu(v) oxidation with its following disproportionation.^23,30^ These small Pu polymers detected by ESI-TOF might be also responsible for the equilibration between the Pu(iii)*/*Pu(iv) and Pu(v)*/*Pu(vi) plutonyl species.^23^

However, to determine exactly if other oxidation states of Pu are present, one needs to use a direct method of probing Pu electronic 5f states at real state conditions. Recently developed synchrotron-based method – X-ray absorption near edge structure (XANES) in high energy resolution fluorescence detection (HERFD) mode^31,32^ – is a powerful technique to investigate Pu species, which allows for the determination of different oxidation state impurities with a high precision (in order of 2%).^33^ We have recently investigated PuO_2_ NPs under other conditions (at pH 8 and pH > 10) by HERFD method and have found that Pu(iv) is the dominating oxidation state for all investigated NPs, synthesized under environmentally relevant and waste storage conditions. The aim of this study is to determine if similar PuO_2_ NPs are formed under acidic conditions and to investigate Pu oxidation states in these NPs. It is also crucial for a colloid investigation to determine the oxidation state distribution both in the solid phase and in the solution, and methods used for this task should be sensitive enough to small impurities of other oxidation states apart from Pu(iv).

Here we report the first investigation of PuO_2_ NPs, synthesized under various pH from 1 to 4 and characterized by high-resolution transmission electron microscopy (HRTEM), selected-area electron diffraction (SAED) and XANES in HERFD mode at the Pu M_4_ edge.

## 2. Experimental

### 2.1 Synthetic procedures

Initial Pu(iv) solution was obtained from Pu(iii) solution by oxidation with NaNO_2_ in 5 M HNO_3_ and verified with UV-vis spectrometry (TIDAS 100 J&M Analytics and UV-800, Shimadzu, see Fig. S1†). The pH of the Pu(iv) starting solution was lower than 1 due to the acid presence. To synthesize NPs, an ammonia aqueous solution was added under continuous stirring to Pu(iv) solution to reach pH 1–4. The total concentration of Pu in the solution was 0.5 × 10^−3^–1 × 10^−3^ M for pH 1 and 0.1 × 10^−3^ M for pH 2–4. The samples were named “Pu(iv) pH *X*”, where *X* is corresponding to the pH. All experiments were conducted under ambient conditions.

Solution composition was investigated with UV-vis spectrometry, spectra were registered from 400 to 900 nm. The kinetics of the precipitation was studied by periodic measurements of Pu concentration in solution.

After 2 hours of synthesis the pH and redox potential (Eh) was measured with pH- and Eh-electrodes (Mettler Toledo), (values are listed in Table S1†).

For HERFD measurements a residue was centrifuged after 2 h of reaction (3900 g, EBA 12 (Hettich), 30 min–2 h). Concentrated samples were not washed from the initial solution to preserve the pH and were afterwards packed to special holders in the form of wet pastes. Samples were sealed in the cells with two layers of kapton foils with a thickness of 25 and 8 μm respectively.

PuO_2_ was a commercial sample (Batch I.D. No. Pu-242-327A1, Oak Ridge National Laboratory, USA), which was characterised previously.^28^ To prepare PuF_4_ and PuF_3_ references, fivefold excess of the hydrofluoric acid HF was added to 2 × 10^−3^ M Pu(iv) and 5 × 10^−3^ M Pu(iii) solutions respectively, solutions were verified with UV-vis spectrometry (Fig. S1†). To prepare Pu(iii) solution initial Pu stock solution was reduced by hydroxylamine hydrochloride when slightly heated in 1 M HClO_4_, while Pu(iv) solution was obtained earlier to prepare NPs.

The size and morphology of the samples were verified by HRTEM recorded with an aberration-corrected JEOL 2100F operated at 200 kV. Samples were dripped in small drops onto the copper grids and let dried.

### 2.2 X-ray absorption near edge structure (XANES) in high energy resolution fluorescence detection (HERFD) mode at the Pu M_4_ edge

XANES spectra in HERFD mode were measured at the MARS beamline at the SOLEIL synchrotron (Saint-Aubin, France).^34,35^ The storage ring was operating in top-up mode at an electron current of 500 mA, 2.5 GeV. Higher harmonic rejection and vertical focusing were achieved using the Si strip of each mirror inserted before and after the DCM with a 4 mrad incidence angle. The incident energy was calibrated using the absorption K-edge of potassium in a KBr pellet (3.6 keV). The incident X-ray flux on the sample position was 1.9 × 10^9^ pH s^−1^ at 3.5 keV. The beam size on the sample was found to be 250 μm × 150 μm FWHM (HxV). HERFD spectra were measured using the crystal-analyser X-ray spectrometer in the Rowland geometry and a KETEK single element silicon solid-state detector. The samples were oriented at 45° with respect to the incident beam. A He-filled chamber was used to reduce the scattering of the emitted X-rays by the air between the sample, the crystal analyser and the detector. Only one Si(220) crystal analyser was used in that experiment. The overall energy resolution of the spectrometer was found to be 1.1 eV (at the 7068 eV) as derived from the FWHM of the elastic scattering peak at the double energy.

To calculate the fractions of different oxidations states of Pu, the iterative transformation factor analysis (ITFA) approach was used.^36^ It was successfully applied to the uranium and Pu compounds studied by HERFD at the U and Pu M_4_ edges.^33,37^ The ITFA approach is implemented as follows: at first, a principle component analysis (PCA) has been done to determine the number of individual components contributing to the spectrum. Then the iterative target test (ITT) procedure was applied to obtain the noise-filtered spectrum of the components. The ITFA analysis shows a relative concentration error in the order of 2%, according to the root mean square error (RMS). More information can be found in ESI.†

### 2.3 Computational details

To obtain the HERFD spectra at the Pu M_4_ edge, the core-to-core (3d–4f) resonant inelastic X-ray scattering (fingerprint) intensity maps were calculated on the emission *versus* incident photon energy scales and a cut at the constant emission energy, corresponding to the maximum of the RIXS intensity was made along the incident photon energy axis. The RIXS maps were calculated as described in ref. 38 and 39. For PuF_3_ and PuF_4_, the crystal field multiplet theory approach was used because the charge-transfer effects, as a result of the Pu 5f – F 2p hybridization, do not contribute significantly to the HERFD spectra due to a large band gap and consequently a large value for the charge-transfer energy. The Slater integrals F^2,4,6^ (5f,5f), F^2,4^ (3d,5f), F^2,4,6^ (4f,5f) as well as G^1,3,5^ (3d,5f) and G^0,2,4,6^ (4f,5f) calculated for the Pu(iii) and Pu(iv) ions were scaled down to 80% of their *ab-initio* Hartree–Fock values in the computation of the RIXS maps. For PuF_3_, the values of Wybourne's crystal field parameters for *D*_3h_ symmetry were adopted from ref. 40 and were set to *B*^2^_0_ = 0.024, *B*^4^_0_ = −0.073, *B*^6^_0_ = −0.214, *B*^6^_6_ = 0.125 in eV. For PuF_4_, the values of the crystal-field parameters for *C*_2v_ symmetry were set to *B*^2^_0_ = 0.140, *B*^2^_2_ = 0.006, *B*^4^_0_ = −0.350, *B*^4^_2_ = 0.383, *B*^4^_4_ = −0.444, *B*^6^_0_ = −0.177, *B*^6^_2_ = 0.157, *B*^6^_4_ = −0.142, *B*^6^_6_ = 0.226 in eV, as derived from the analysis of the optical absorption spectra.^41^ The ground, intermediate and final states of the spectroscopic process were represented by the 3d^10^5f^*n*^, 3d^9^5f^*n*+1^ and 4f^13^5f^*n*+1^ configurations, respectively, where *n* = 5 for Pu(iii) and *n* = 4 for Pu(iv).

Since the inclusion of the charge-transfer effects is important for the description of the high-energy spectroscopic data of PuO_2_, (see *e.g.*^42^), the Pu 3d-to-4f RIXS map was calculated in the framework of the Anderson impurity model (AIM).^43^ The model parameter values were chosen to be the same as in previous calculations^37^ and their values were as follows: energy for the electron transfer from the O 2p band to the unoccupied Pu 5f states *Δ* = 0.8 eV; 5f-5f Coulomb interaction *U*_ff_ = 5.7 eV; 3d(4f) core hole potential acting on the 5f electron *U*_fc_ = 6.5(6.0) eV and Pu 5f – O 2p hybridization term *V* = 1.1 eV (0.9 eV) in the ground (intermediate and final) state of the spectroscopic process. A linear combination of the 4f^4^ and 4f^5^
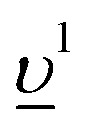
 configurations was used to describe the ground state of the spectroscopic process and intermediate (final) state was represented by a combination of 3d^9^5f^5^ and 3d^9^5f^6^
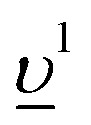
 (4f^13^5f^5^ and 4f^13^5f^6^
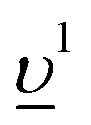
), where 
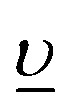
 stands for an electronic hole in the O 2p band. The *ab-initio* values of the Slater integrals obtained for Pu(iv) using the Hartree–Fock formalism were scaled down to 80% to account for the solid state effects. Wybourne's crystal field parameters for cubic symmetry were set to *B*^4^_0_ = −0.93 eV and *B*^6^_0_ = 0.35 eV.

## 3. Results and discussion

**Fig. 1:**
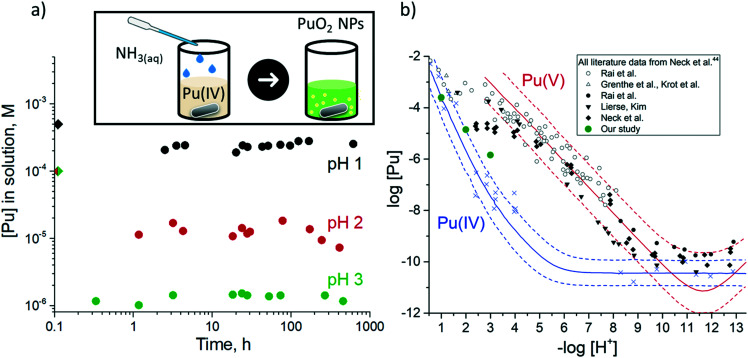
a) Pu concentration in solution (supernatants after centrifugation) during the PuO_2_ synthesis at different pH. The rhombuses at 0 h are the initial Pu concentrations. Inset: The scheme of the synthetic route, b) Solubility of PuO_2_ at 20–25 °C as a function of [H^+^] in the presence of oxygen compared to the Pu(iv) and Pu(v) solubility data and literature data: white and black symbols represent the total plutonium concentration under air and argon atmosphere respectively, blue crosses – a fraction of Pu(iv) found in literature data^44^ and determined by solvent extraction technique. Calculated solubilities of PuO_2_(am) and PuO_2+*x*_(am) are shown as blue and red lines respectively.^44^

We have recently studied the formation of PuO_2_ NPs at pH 8 and >10 and we have used the total Pu concentration of 6 × 10^−5^ M, which exceeds its solubility in line with the available thermodynamic prediction.^18,28,44^ According to available solubility data (Fig. 1b), at pH < 4 precipitate cannot form at this concentration of Pu.^44^ Therefore we have decided to use 1 × 10^−4^ M Pu concentration. According to thermodynamic information, this concentration is still not sufficient for Pu precipitation; nevertheless, the residue is formed at pH 4 and even pH 2 (which is remarkable). An even higher Pu concentration of 1 × 10^−3^ M is used to obtain PuO_2_ NPs at pH 1. The instant decrease of the Pu solution concentration unequivocally confirms that precipitate forms during the first minutes of the reaction; simultaneously Pu concentration of the solution is established at a certain level (Fig. 1a). The steady-state concentration of Pu in solution is influenced by pH and correlated with the solubility control of the NPs formation process. It is known, that Pu solubility decreases with the increase of pH^17,18,21^ and our results reproduce this trend well, however, the absolute values of the Pu concentration are not consistent with previous results (Fig. 1b). Regardless of this disparity, it is quite uncommon that NPs are still formed even at pH 1 under these conditions, though it has been shown previously that it is possible at higher Pu concentrations upon heating.^45^

### 3.1 Characterization of the PuO_2_ NPs formed at low pH

First, we have investigated structural and electronic properties of the formed PuO_2_ NPs with HRTEM and HERFD methods.

**Fig. 2:**
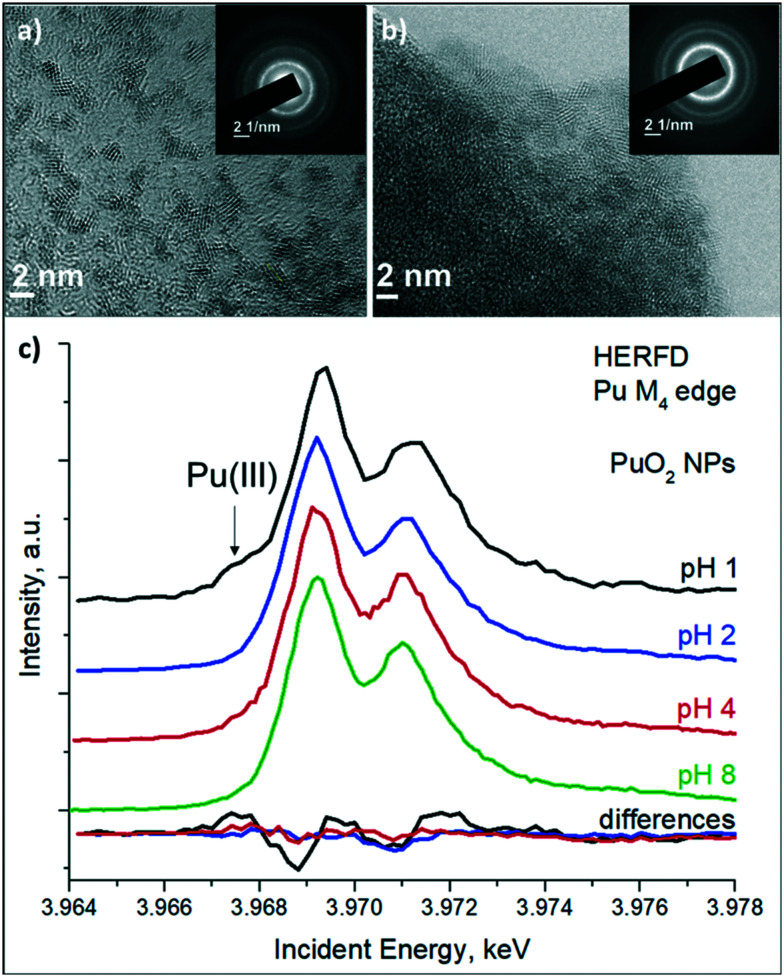
Solid phase characterisation: HRTEM data for NPs from Pu(iv) solution at a) pH 1, b) pH 2. Inset: Corresponding electron diffraction patterns, c) Pu M_4_ HERFD spectra from NPs samples. Spectral difference between Pu samples and Pu reference “Pu(iv) from pH 8” are shown at the bottom.^28^

The HRTEM data reveal that independent from pH conditions, small crystalline NPs are formed with an average particle size of 2 nm, as reported in Fig. 2a and b and Table S2.† Electron diffraction patterns for the samples (Fig. 2a and b insets) confirm a fluorite crystal structure similar to bulk PuO_2_. Thus, we conclude that NPs formed during fast chemical precipitation from Pu aqueous solutions are the same for acidic and alkaline pH conditions, *i.e.* 2 nm crystalline NPs with a structure similar to bulk PuO_2_. However, at pH 1 particles are less agglomerated. One can see separated particles in the HRTEM's grid (Fig. 2a), while for pH 2 sample (Fig. 2b) and pH 8 and >10 they are mainly presented as agglomerates (Fig. S2†).^28^ However, it should be taken into account, that deep vacuum conditions of HRTEM may have an impact on the NPs, therefore the implementation of non-destructive HERFD method is favourable.

HERFD method also shows resemblance among the three investigated PuO_2_ NPs samples, formed at pH 1, 2 and 4 (Fig. 2c). HERFD method at the Pu M_4_ edge is a highly effective method of oxidation state identification. Spectra recorded on Pu systems can be straightforwardly analysed by a fingerprint approach.^28,33,37,46^ They are compared to the spectrum of PuO_2_ NPs obtained from Pu(iv) at pH 8, which was confirmed to have a PuO_2_-like structure and therefore is used as a reference in this study.^28^ Inspection of Fig. 2c shows that spectra of NPs at pH 2 and 4 are identical to that of the reference while spectral features for NPs at pH 1 are broader and there is also a shoulder on the left side from the main edge (at ∼3967 eV). The characteristic spectral difference for all PuO_2_ NPs compounds at various pH is shown at the bottom of Fig. 2c. The low energy shoulder in the X-ray spectroscopy process is generally attributed to a change towards a lower oxidation state and can indicate the presence of Pu(iii) in the case of PuO_2_ NPs formed at pH 1. The peak broadening of the absorption feature at the ∼3971.5 eV indicates the presence of higher oxidation states (Pu(vi), most likely). The broadening of the main edge remains the same (as can be seen from the spectra directly or by checking the difference curve at this energy range) therefore contribution of Pu(v) can be excluded. Generally, the Pu(v) M_4_ HERFD has a special energy position, which is shifted by 0.6 eV from the Pu(iv).^28,37^ Therefore it allows us to suggest that PuO_2_ NPs formed at pH 1 contain the mixture of the Pu(iii), Pu(vi) and Pu(iv) oxidation states. It should be noted that even well-known Pu(iii) compounds have never been studied before by the HERFD at the Pu M_4_ edge.

**Fig. 3:**
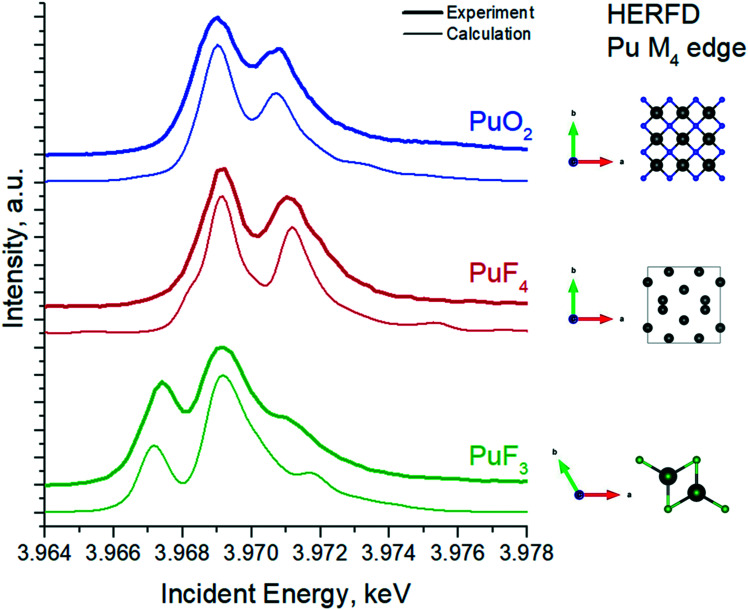
Experimental and calculated Pu M_4_ HERFD spectra of PuO_2_, PuF_4_ and PuF_3_ samples. Inset schematically represents crystal structures of the compounds.

As such, we have recorded experimental data on the PuF_3_ reference to verify the position and the shape of the HERFD features at the Pu M_4_ edge (Fig. 3). Surprisingly, two intense peaks at ∼3967 eV and ∼3969 eV in the Pu M_4_ HERFD are detected, which might indicate the presence of the Pu(iii) and Pu(iv) oxidation states.^39^ However, the calculation of the Pu M_4_ HERFD spectrum shows that both intense peaks are associated with transitions due to the Pu(iii) oxidation state. This result is very remarkable and should be taken into account in the next studies of any Pu(iii) systems by the Pu M_4_ HERFD method. Thus our assumption that the spectrum of PuO_2_ NPs formed at pH 1 has Pu(iv) as a dominating oxidation state with the additional presence of Pu(iii) is confirmed.

Based on the theoretical achievements and experimental data, recorded for the Pu model systems, we have estimated the exact contribution of the Pu(iii), Pu(iv) and Pu(vi) oxidation states in the Pu M_4_ HERFD data for the PuO_2_ NPs formed at the pH 1, pH 2 and pH 4 with the ITFA package.^36,47^ It turns out that PuO_2_ NPs formed at pH 1 contain 10% for Pu(iii), 80% of Pu(iv) and 10% for Pu(vi). The contribution of Pu(v) has not been detected (c.f. Fig. S3†). The equivalence of concentrations suggests that Pu(iii) and Pu(vi) are likely originated from the solution, formed by Pu(iv) disproportionation reaction. Nonetheless, Pu(iv) remains the dominating oxidation state and is found to be in the order of 80%. It should be noted here that the Pu(iv) signal originates from both solution and PuO_2_ NPs. For the PuO_2_ NPs, synthesized at pH of 2 and 4, the main edge is observed at 3969 eV, indicating that both compounds are in the pure Pu(iv) oxidation state. No Pu(iii), Pu(v) and Pu(vi) contributions have been found by ITFA for both compounds (Fig. S3†). The characteristic spectral difference for all PuO_2_ NPs compounds at various pH, shown at the bottom of Fig. 2c confirms ITFA analysis.

As a first conclusion, we have found by HERFD and HRTEM that NPs obtained from pH 1, 2 and 4 are very similar to PuO_2_ NPs obtained from alkaline pH: identical crystal structures and Pu(iv) as the dominating oxidation state in all NPs. However, HRTEM shows that PuO_2_ particles formed at pH 1 are less agglomerated. This indicates the possible increase of NPs stability in solution as well and complicates the process of particle separation from the solution, requiring high-speed centrifuges or ultrafiltration.^48^ By reason of instrumental limitations during sample preparation caused by Pu-handling restrictions, we cannot exclude the presence of a mother liquid used to transfer minor amounts of solid-phase samples at pH 1 for the HERFD measurements. To answer the most confusing question of why Pu(iii) and Pu(vi) are present in the HERFD spectrum of this sample, we have examined the presence of various Pu species in the solution and their evolution over time by UV-vis spectroscopy. It might help to verify whether other oxidation states originate from the mother liquid or the PuO_2_ NPs themselves contain Pu(iii) and Pu(vi) species. However, the absence of differences in HRTEM and the PuO_2_ – like structure according to SAED are already strong arguments against the statement that NPs contain Pu different from Pu(iv). Therefore, as a next step, we have studied the evolution of the initial Pu solution over time.

### 3.2 Evolvement of the initial Pu solution over time

**Fig. 4:**
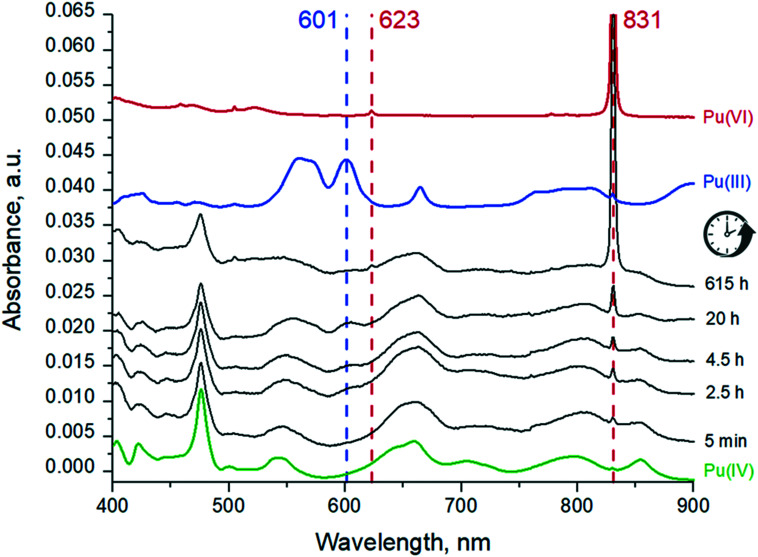
UV-vis spectra of Pu(iv) pH 1 sample solution after different times of reactions and reference solutions with wavelengths of the characteristic peaks.

UV-vis spectra of the Pu solution recorded at different time intervals are shown in Fig. 4. At the beginning of the reaction, the Pu(iv) is the dominating oxidation state with traces of Pu(vi) (c.f. peaks at 831 and 623 nm), while eventually the Pu(iv) concentration decreases and significant amounts of Pu(iii) (the peak at 601 nm) appear. Due to the substantially different molar extinction coefficients of Pu(iii) peak at 601 nm and Pu(vi) peak at 831 nm (38 and 555 respectively), the concentration of Pu(iii) after 20 h is actually higher than Pu(vi) while the peak at 601 nm is less intense.

**Fig. 5:**
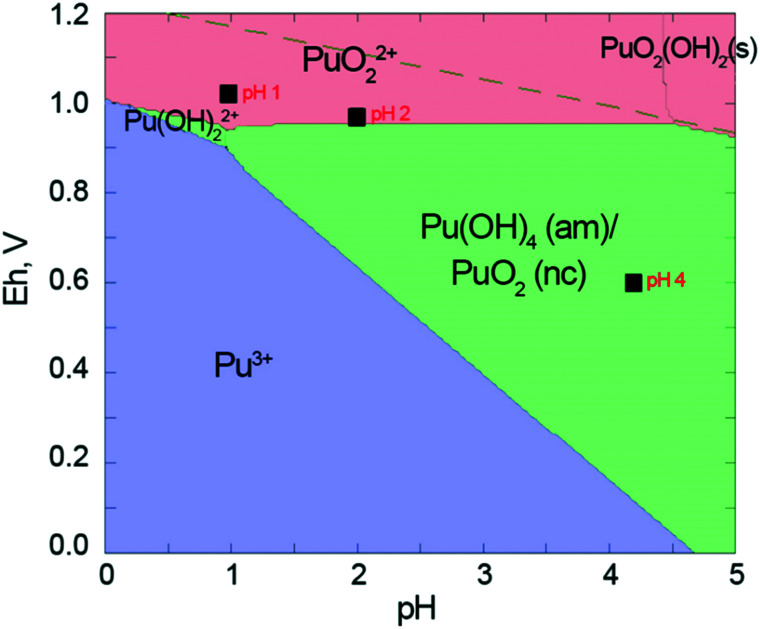
Pourbaix diagrams for Pu, calculated in MEDUSA software ([Pu] = 5 × 10^−4^ M) together with experimental data on pH–Eh conditions of the synthesis. The thermodynamic data from NEA database are used.

Pu concentrations are calculated from UV-vis data using the Beer–Lambert law. After 20 hours of interaction (which corresponds to the time needed for the preparation of the samples for HERFD), the distribution of Pu oxidation states in solution is as follows: Pu(IV) is 1.4 × 10^−4^ M, Pu(iii) is 0.4 × 10^−4^ M and Pu(vi) is 0.09 × 10^−4^ M. Pu(v) contribution was not detected by UV-vis spectrometry. Interestingly, the Pu(iii) concentration decreases after 20 h while Pu(vi) increases. Since the Pu(iii)/Pu(vi) ratio changes but the total Pu concentration in solution remains constant, it leads to the conclusion that redox reactions are still occurring in the system (Fig. S4†). We continued to investigate the solution over time and found that after 11 months, Pu(iv) concentration in the solution decreased by half, while Pu(vi) concentration doubled (Fig. S4†). The concentration of Pu(iii) can not be detected at this stage by UV-vis spectrometry, but the total Pu concentration in the solution remains the same. We believe that the oxidation of Pu(iii) and Pu(iv) is caused by the presence of atmospheric oxygen. Moreover, the evaluation of the pH and Eh conditions (Fig. 5) indicates that Pu(vi) is thermodynamically stable in this region, therefore, Pu(iii) and Pu(iv) eventually oxidize to Pu(vi).

Summarizing the results of solid and solution characterisation, we conclude that Pu oxidation states other than the Pu(iv) observed in the HERFD spectrum of the sample at pH 1 come directly from the solution since the amount of dissolved Pu is enough to be detected. Samples for HERFD measurements were prepared as wet pastes hence some solution is caught during sample preparation. In the case of PuO_2_ NPs from pH 1, the steady-state concentration of Pu is higher (Fig. 1a), allowing a liquid phase contribution to be detected. Moreover, the HERFD method can be used to study all matters (liquids, solids and gases) and the detection limit of the HERFD method is so superior that even a tiny Pu concentrations still present in the solution is detectable. This is the case for the PuO_2_ NPs sample, formed at pH 1, where the HERFD spectrum is a superposition of signals. Moreover, the Pu(iv) signal here recorded on both the solution and PuO_2_ NPs themselves.

### 3.3 Discussion

It is essential to discuss the discrepancies in steady-state solution concentrations of Pu for this study and the one reported in the literature previously (Fig. 1b). There are several factors that might be potentially responsible.

Unlike this study, in previous solubility investigations, the morphology and crystallinity of the particles have not been studied in detail thus these parameters may invoke diverse solubility values. Neck and co-authors^18^ investigated the thermodynamic stability of PuO_2_ and linked particle size and solubility with the Schindler equation. It draws a relation between experimental solubility products (colloid, amorphous and crystalline PuO_2_) and the corresponding particle size. Enhanced solubility of Pu(iv) may be interpreted as an effect of particle size on the Gibbs energy of the small fractions, recorded on both the solid and liquid phases. Therefore, it allows us to conclude that all PuO_2_ NPs themselves, made at different pH levels in the range of 1 to >10, contain only Pu(iv) oxidation state.

In addition, NPs at low pH stabilize in the solution hence in case of insufficient separation may cause systematic solubility overstatement. NPs stability is related to agglomeration reactions, which are controlled by the colloid surface charge and the solution composition.^49^

Besides that, Neck with co-authors^18^ and Rai with co-authors^21^ have suggested that Pu(v)/Pu(vi) is present in solution and it is considered in the thermodynamic model (Fig. 5). Neck and co-authors^18^ investigated solubility and redox reactions of Pu(iv) hydrous oxide and found, that solubility is controlled by hydrous PuO_2+*x*_ (s, hyd) and mixed valent (Pu^V^)_2*x*_(Pu^IV^)_1−2*x*_O_2+*x*_ (s, hyd) solid phases. Rai and co-authors^21^ showed that at pH 1–3 redox potentials are controlled by the oxidative dissolution of amorphous PuO_2_ (am, hyd) and the redox equilibrium between PuO_2_^+^ and PuO_2_^2+^:PuO_2_(am, hyd) ⇆ PuO_2_^+^ + e^−^PuO_2_^+^ ⇆ PuO_2_^2+^ + e^−^Based on this, the appearance of Pu(iii) in the solution just after the formation of the solids might be surprising (Fig. 5). Despite the assumption that Pu(iii) does not contribute much to the Pu reactions at ambient conditions (with a few exceptions though^23,50^), our results undoubtedly confirm its presence in the solution.

We consider Pu(iv) disproportionation to Pu(iii) and Pu(vi) as the main mechanism of Pu(iii) formation at pH 1. Due to the thermodynamical instability of Pu(iii) and Pu(iv) under such pH and Eh conditions, they will eventually oxidize to Pu(vi) as observed in this study. It should be noted that after 615 h and then 11 months the Pu(vi) fraction has increased significantly (Fig. 4 and S4†), testifying that redox reactions occur during this period.

## 4. Conclusions

In this work, we explore in detail the formation of the PuO_2_ NPs at acidic conditions and compare it with NPs formed in alkaline media. It was shown that NPs formed at pH 1–4 have a PuO_2_-like structure according to HRTEM data. Taking into account our previous investigations,^28,37^ we show that for the wide pH range from 1 to >10 small crystalline PuO_2_ NPs are formed with an average size of 2 nm. The dominating oxidation state for the particles is proven to be Pu(iv) with a help of the HERFD method at the Pu M_4_ edge in combination with electronic structure calculations by AIM.

We demonstrate here that at pH 1 a stable Pu colloid is formed with a significant amount of Pu in solution. Plutonium in acidic solutions is eventually present at several oxidation states: Pu(iii), Pu(iv), Pu(vi). Nevertheless, the solid phase remains intact during these processes, though Pu from solution may misrepresent results due to the exceptional stability of obtained colloids. Therefore, the contribution of Pu(iii) and Pu(vi), observed in the HERFD spectrum of PuO_2_ NPs at pH 1, originates from the solution rather than from the NPs themselves. It has been detected due to the high sensitivity of the HERFD method towards the Pu concentration at these conditions.

Furthermore, we report here the first experimental HERFD data on the PuF_3_ compound recorded at the Pu M_4_ edge. Theoretical calculations confirm that the spectral shape of Pu(iii) M_4_ HERFD spectrum looks very different from Pu(iv) due to the ground state configuration with 5f^3^ character and the local structure near Pu. The unusual spectral shape of the Pu M_4_ HERFD on Pu(iii) should be taken into account in the next studies of any Pu(iii) systems by that method.

We believe that PuO_2_ NPs formation at low pH is very specific due to the high colloid stability under these conditions as well as relatively significant solubility. The difficulties of solid and liquid phase separation may lead to misinterpretation of solubility and characterization results. Stable agglomerates maintain the sustainability of Pu colloids and their behaviour is fundamentally different from simple aqueous solution. All specific properties of such systems must be carefully considered for technological processes where Pu colloid is involved. Overall we believe that the results from this study are significant for applied, fundamental and environmental science.

## Author contributions

K. O. K. A. Yu. R. and S. N. K. planned and supervised the project. E. G., A. Yu. R., A. K. and S. W. performed the synthesis. E. G., M. H., S. B. and K. O. K. performed HERFD experiments at the Pu M_4_ edge. A. E. performed HRTEM characterization of the samples. S. M. B. performed Pu 3d–4f RIXS and Pu M_4_ HERFD calculations. K. O. K., E. G., A. Yu. R., and S. N. K. co-wrote the paper. All authors discussed the results and contributed to the final manuscript.

## Conflicts of interest

There are no conflicts to declare.

## Supplementary Material

EN-009-D1EN00666E-s001
