# Supplementary material for: To form or not to form: PuO2 nanoparticles at acidic pH
Source: Environ Sci Nano. 2022 Mar 11;9(4):1509–18. doi: 10.1039/d1en00666e (PMC9009106; doi:10.1039/d1en00666e)
Supplement: EN-009-D1EN00666E-s001 [file EN-009-D1EN00666E-s001.pdf]

Electronic Supplementary Material (ESI) for Environmental Science: Nano.
This journal is © The Royal Society of Chemistry 2022

Supplementary information

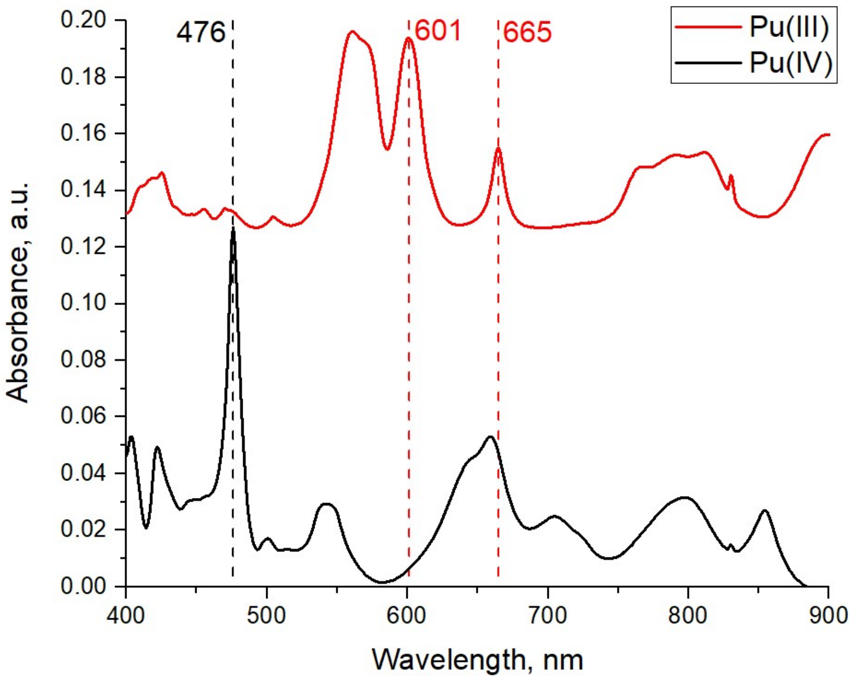

Fig. S1. UV-vis spectra of Pu initial solutions with wavelengths of the characteristic peaks.

Table S1. The pH and Eh values after the end of the reactions.

**Sample**	**pH**	**Eh, mV**
Pu(IV) pH 1	1.0	790
Pu(IV) pH 2	2.1	470
Pu(IV) pH 4	4.2	390

Table S2. The average size of the NPs according to HRTEM data. For each sample, 100-150 measurements have been done.

Sample	Size, nm
Pu(IV) pH 1	2.3±0.4
Pu(IV) pH 2	2.7±0.6
Pu(IV) pH 4	3.4±0.8

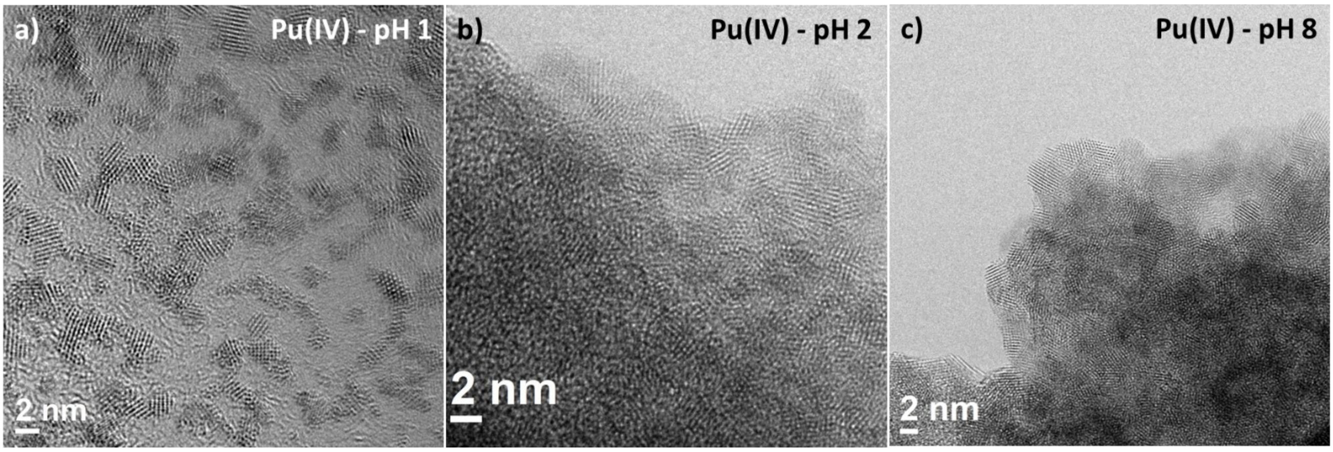

Fig. S2. HRTEM images of NPs from a) Pu(IV) pH 1, b) pH 2, c) pH 8.

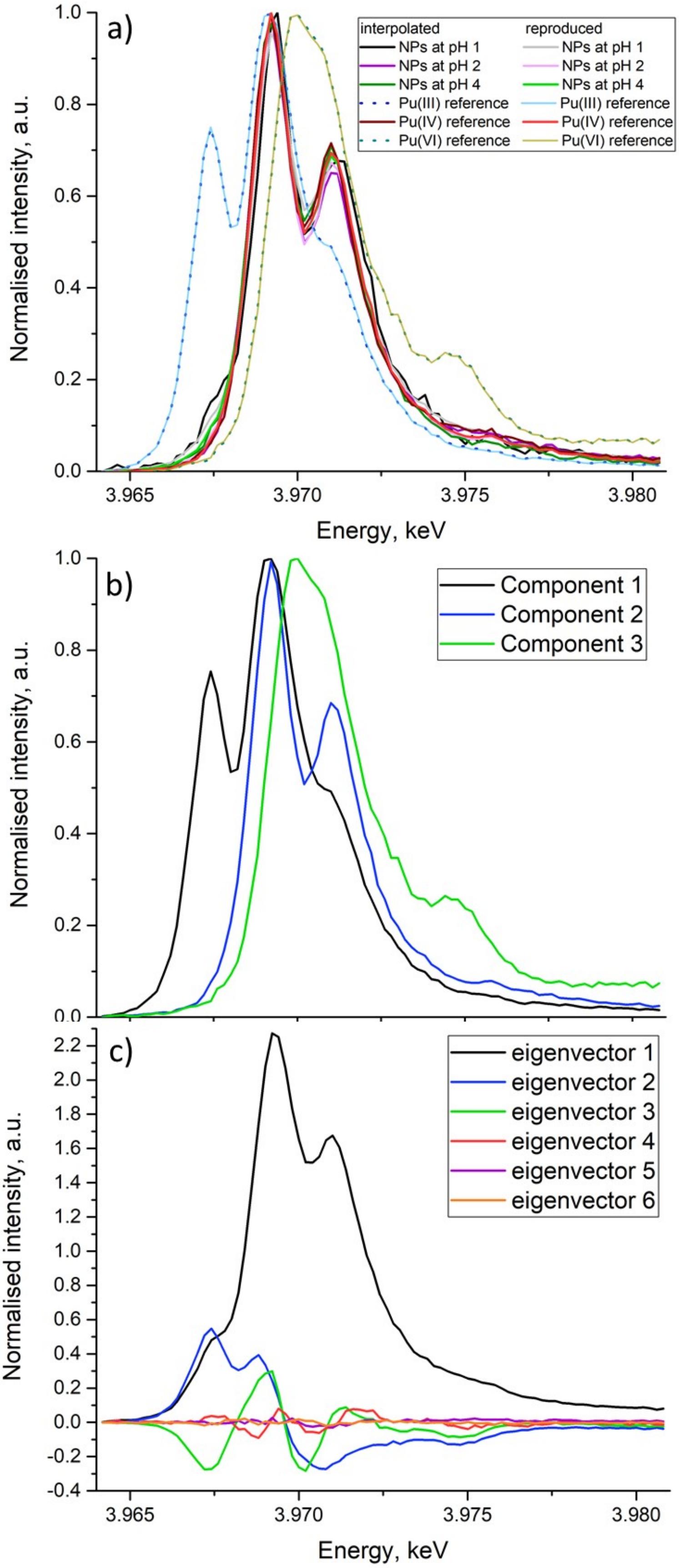

Fig. S3. ITFA results: a) Experimental spectra of samples and references and their reproduction by principal components, b) ITT-extracted principal components, responsible for the data reproduction, c) Isolated eigenvectors, reproducing data and determining the number of different contributions using eigenanalysis.

Iterative transformation factor analysis (ITFA): The Pu(III), Pu(IV) and Pu(VI) contributions were derived for HERFD data for three samples: nanoparticles from Pu(IV) solution at pH 1, 2 and 4. The following compounds for the Pu(III), Pu(IV) and Pu(VI) references have been used: PuF_3, PuO_2 NPS at pH 8, $K_4PuO_2(CO_3)_2$, respectively. The experimental and reproduced spectra are shown in Fig. S3a. The number of principal components needed to reproduce spectra was determined with principal component analysis (PCA), (c.f. Fig. S3c). One can see, that only three eigenvectors contribute to the signal, while eigenvectors 4-6 show only experimental error (i.e. spectral noise) and can be neglected. The iterative target test (ITT) procedure was used in order to find the relative concentrations of the three components for spectra of NPs. It was found, that sample «NPs at pH 1» contains 10% of Pu(III), 10% of Pu(VI) and 80% of Pu(IV) while «NPs at pH 2» and «NPs at pH 4» contain 100% of Pu(IV).

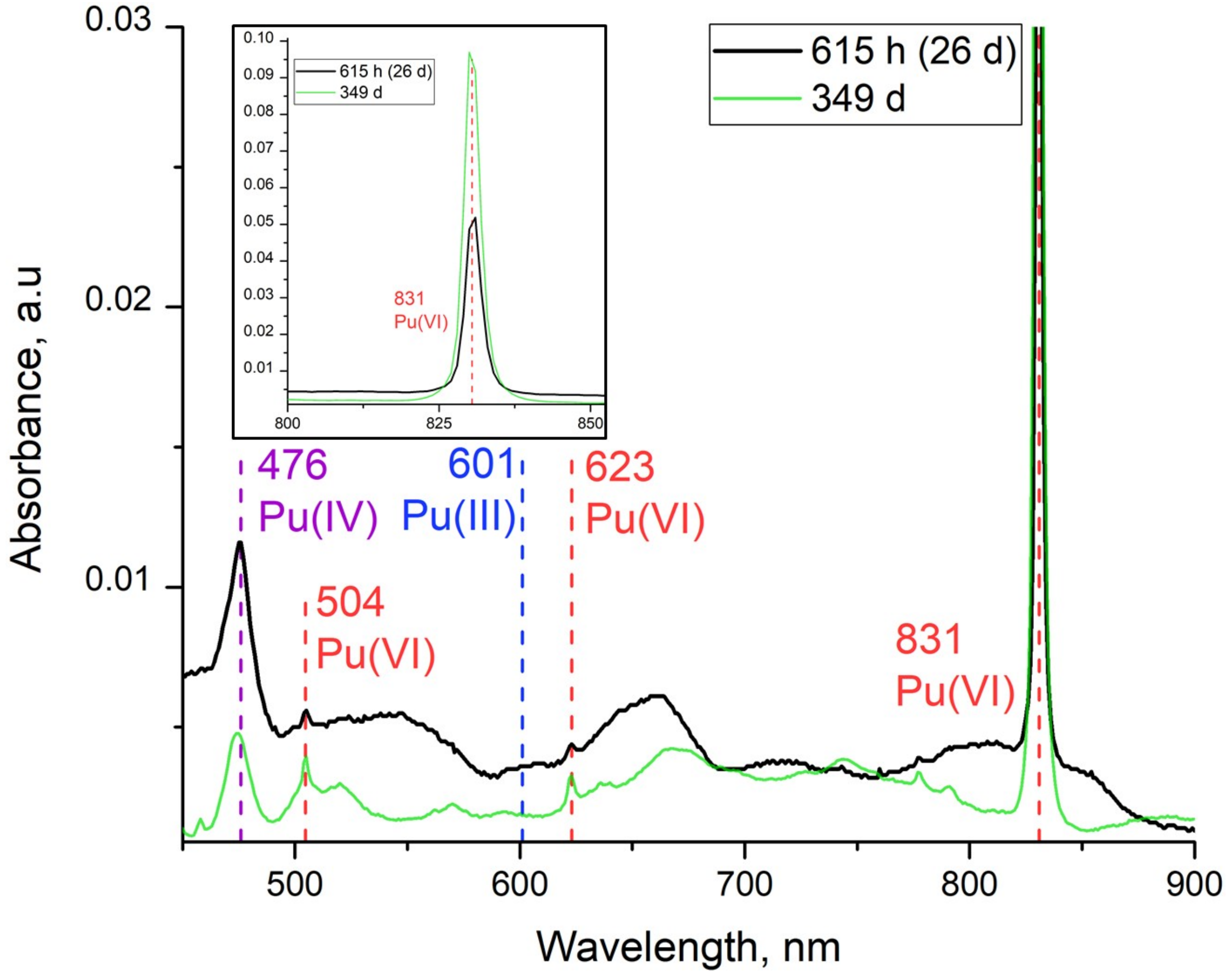

Fig. S4. UV-vis spectra illustrating the oxidation state distribution of Pu solution. Dotted lines show wavelengths of the characteristic peaks. Inset: the peak of Pu(VI) at full intensity.
